# Wie kann wissenschaftliches Arbeiten in der Medizin vermittelt werden? Digitale Lehre in Zeiten der COVID-Pandemie am Beispiel der HNO-Heilkunde

**DOI:** 10.1007/s00106-022-01158-w

**Published:** 2022-03-16

**Authors:** A. K. Rauch, C. Offergeld, Manuel Christoph Ketterer

**Affiliations:** Klinik für Hals, Nasen, Ohrenheilkunde, Universitätsklinikum Freiburg, Medizinische Fakultät, Albert-Ludwigs-Universität Freiburg, Killianstr. 5, 79106 Freiburg, Deutschland

**Keywords:** Datenkompetenz in der Medizin, Digitale Lehre, Wissenschaftliches Arbeiten, Kompetenzbasierte Lehre, COVID-19-Pandemie, Data literacy in medicine, Digital education, Scientific education, Competence-based education, COVID-19 pandemic

## Abstract

**Hintergrund:**

Um aus der Not der Umstellung der Lehre in Zeiten der COVID-Pandemie eine Tugend zu machen, entwickelten wir das Konzept eines neuen digitalen Seminars zum wissenschaftlichen Arbeiten. Digitale Kompetenz begründet den kompetenten Umgang mit Daten in medizinischer Lehre und wissenschaftlicher Ausbildung. Diese Studie präsentiert die Ergebnisse der studentischen Lehrevaluation des Seminars mit Fokus auf den Erwerb von digitaler und wissenschaftlicher Kompetenz.

**Methode:**

In diese prospektive Fragebogen-Studie wurden 265 Studierende eingeschlossen. Das Seminar beinhaltete eine Einführung über die Kriterien guten wissenschaftlichen Arbeitens, gefolgt von einer individuellen Arbeitsphase der Studenten mit Bearbeitung einer wissenschaftlichen Publikation und selbstständigen Erstellung des zugehörigen Abstracts mit abschließender Evaluation.

**Ergebnisse:**

Das Seminar wurde insgesamt gut bewertet. In Freitext-Kommentaren wurde deutlich, dass sich die Studierenden statt digitaler Lehre dennoch Anwesenheitsseminare zum Thema wünschten. Die Studierenden gaben an, dass ihre wissenschaftliche Kompetenz durch das digitale Seminar und das selbständige Verfassen eines Abstracts verbessert wurde.

**Schlussfolgerung:**

Die digitale Lehre wurde von den Studierenden zwar nicht ausschließlich positiv bewertet, verbesserte jedoch deren subjektive wissenschaftliche Kompetenz und erfüllte deren Wunsch einer digitalen Transformation der Lehre und damit auch die Ziele des neuen Nationalen Kompetenzbasierten Lernzielkatalogs der Medizin.

## Abstract (en)

**Introduction:**

To make a virtue out of necessity by establishing a digital teaching curriculum in otorhinolaryngology (ORL) during the COVID-19 pandemic, the authors created the concept of a new digital scientific seminar. Digital competence forms the basis for data literacy in medical and scientific education. In this study, the students’ evaluation of the seminar focusing on digital and scientific skill acquisition is presented.

**Methods:**

Included in this prospective monocentric questionnaire study were 265 students. The seminar started with an introduction on the criteria of a good publication, followed by the individual task of understanding the publication on a main ORL topic and writing its abstract. After the seminar, students completed the evaluation questionnaire.

**Results:**

Overall, results showed that students rated the seminar well. Free-text comments added that although live teaching was preferred, students found their digital and scientific competence increased through the task of writing a publication abstract on their own.

**Conclusion:**

Digital education was not only rated well, but improved the students’ subjective scientific competence, satisfied the students’ wish for digital transformation, and likewise fulfilled the national goals of competence-based education.

## Hintergrund

Durch das neuartige Coronavirus [27] musste die Lehre in der Medizin innerhalb von kürzester Zeit in ein vollständig digitales Curriculum umgewandelt werden [21]. Die COVID-19-Pandemie stellt nicht nur eine individuelle persönliche Herausforderung dar. Sie stellt auch insbesondere die Lehre in der Medizin, die neben theoretischen auch praktische Fähigkeiten vermittelt, vor eine völlig neue Aufgabe. Ab dem Sommersemester 2020 wurden alle Studierenden in der Hals-Nasen-Ohren-Heilkunde vollständig online unterrichtet. Die medizinische Lehre steht vor den Herausforderungen der Digitalisierung sowie der kompetenzorientierten curricularen Neuausrichtung. Kennzeichnend für die Entwicklung in der Medizin und in der medizinischen Lehre ist dabei das abnehmende Interesse an wissenschaftlichem Arbeiten und dem bekannten sinkenden Anteil an wissenschaftlichem Nachwuchs unter den Medizinern sowohl international [9] als auch insbesondere in Deutschland mit hohen Drop-out-Raten vor allem nach der Promotion [15]. Das Ziel unseres Seminars und unserer Studie bestand darin, bei Medizinstudierenden die Motivation zur wissenschaftlichen Betätigung durch ein neuartiges digitales Seminarkonzept mit HNO-Fokusthemen zu fördern.

### Kompetenzbasierte Lehre in der Medizin

Dem Canadian-Medical-Education-Directives-for-Specialists-Rollenmodell entsprechend, wurde der Nationale Kompetenzbasierte Lernzielkatalog Medizin in Deutschland mit dem Ziel einer ergebnisorientierten Reorganisation des Medizinstudiums implementiert [7]. Digitalisierung hat nicht nur durch die Pandemie, sondern allgemein auch im Alltag und Arbeitsleben zunehmend an Bedeutung gewonnen. Das Erlernen digitaler Kompetenzen besitzt einen hohen Nutzengrad [14]. Dies stellt einen zusätzlichen Impuls dar, digitale Kompetenzen in die medizinische Lehre zu integrieren und so dem Ziel einer kompetenzbasierten Lehre zu folgen.

### Abnehmendes Interesse an wissenschaftlichem Arbeiten von Medizinstudierenden

Offiziellen Angaben zufolge ist das Interesse an wissenschaftlichem Arbeiten unter Medizinstudierenden in Deutschland abnehmend [15, 18]. Weiterhin ist eine Abwanderung von Ärzten v. a. in die Schweiz, nach Österreich und in die USA zu verzeichnen – laut Statistik der Bundesärztekammer (Stand 2018) [5], und die Gefahr einer zunehmenden Abwanderung von forschenden Ärzten insbesondere zum als attraktiver angesehenen hochschulmedizinischen Forschungsstandort USA [6]. Dem traditionellen deutschen Medizincurriculum wird eine mangelnde Verbindung zum wissenschaftlichem Arbeiten bescheinigt [18] bei gleichzeitig zunehmend höherer Bedeutung von Letzterem [3]. Darüber hinaus sind wenige Karrieremöglichkeiten in der medizinischen Wissenschaft zu verzeichnen [15, 18]. Dies diente uns als Motivation zur Entwicklung eines innovativen Seminars zum wissenschaftlichen Arbeiten innerhalb unseres etablierten HNO-Curriculums.

### Herausforderungen der digitalen Lehre in der COVID-19-Pandemie

Zahlreiche Probleme waren hinsichtlich der schnell benötigten Umstellung auf digitale Lehre in der Pandemie ersichtlich: das Fehlen von digitaler Infrastruktur [8, 14, 21], das Fehlen von etablierten digitalen Kompetenzen im regulären Lehrcurriculum [14, 21] und die grundsätzliche Problematik, praktische Fertigkeiten digital zu lehren [1, 25]. Daher fokussierten wir uns in der Gestaltung unseren Seminars auf den Bereich des wissenschaftlichen Arbeitens und des digitalen Umgangs mit wissenschaftlichen Daten, was eine notwendige Kompetenz für jeden Arzt darstellen sollte.

### Die Evaluation definiert die Qualität der Lehre in der Medizin

Derzeit ist die Qualität der deutschen Lehre in der Medizin durch Standards der „Association for Evaluation“ [26] festgelegt, welche mit den britischen und amerikanischen Standards vergleichbar sind. Dennoch werden Investitionen meist in Wissenschaft und Klinik und nicht in Anschaffungen in der Lehre getätigt [2]. Weiterhin sind die Anerkennungen von Lehrexzellenz aufgrund fehlender Standardisierung und Validierung erschwert. Das steht im Kontrast dazu, dass bereits 2001 84 % der medizinischen Fakultäten in Deutschland Lehrevaluationen durchführten [22]. Vor diesem Hintergrund der zunehmenden Bedeutung der Lehrevaluation entschieden wir uns dazu, diese zielgerichtet für die Bewertung unseres neugestaltenten Seminars zum wissenschaftlichen Arbeiten einzusetzen.

### Das Freiburger Konzept digitaler HNO-Lehre und die Einführung eines neuen wissenschaftlichen Seminars

Das Freiburger HNO-Curriculum wurde in ein digitales Lehr-Curriculum umgewandelt, welches aus fünf Vorlesungen via Zoom, Seminaren als Podcast, Liveseminaren via Zoom, Online-Live-HNO-Untersuchungsunterricht, chirurgischer Lehre mittels Op.-Videos statt Assistenzen im OP, digitaler Ambulanzlehre und digitalen HNO-Lernprogrammen bestand [16]. Ein vorangegangenes Seminar führte die Studierenden in die Datenkompetenz der HNO-Heilkunde ein [20] und wurde sehr positiv bewertet.

### Ziel des Seminars und dieser Studie

Unser neues wissenschaftliches Seminar wurde ab dem Beginn der Pandemie im April 2020 durchgeführt und die Evaluation bis zum Ende des Wintersemesters 2020/2021 ausgewertet (März 2021). Für die Evaluation nutzten wir eine Sechs-Punkte Skala und Freitext-Kommentare [22]. Die Hypothese war, dass das wissenschaftliche Interesse der Studierenden mit ihrer Bewertung des Seminars positiv korreliert und das Seminar deren wissenschaftliches Interesse und deren wissenschaftliche Kompetenz steigert. Grundlegend wird durch wissenschaftliches Arbeiten kritisches Denken gefördert, welches wiederum eine notwendige Kompetenz jeder weiteren professionellen Aktivität ist: Konkret sind Verstehen, Interpretieren und Bewerten von wissenschaftlichen Daten und Publikationen erforderliche Kompetenzen für Ärzte und sollten durch die medizinische Lehre gefördert werden. Daher war das Ziel des Seminars, den Studenten sowohl digitale als auch wissenschaftliche Kenntnisse zu vermitteln. Die vorliegende prospektive monozentrische Studie bewertet den Erfolg unseres digitalen wissenschaftlichen Seminars anhand der studentischen Lehrevaluation.

## Methode

### Studienkollektiv

Alle im Sommersemester 2020 (04/2020–08/2020) und im Wintersemester 2020/2021 (10/2020–02/2020) eingeschriebenen und der HNO-Lehre zugeteilten Studierenden wurden zur Studienteilnahme eingeladen. Die Studie wurde von der Ethikkommission (Nr. 196/20) nach den Kriterien der Deklaration von Helsinki (Version: 2013) der Albert-Ludwigs-Universität Freiburg genehmigt. Alle teilnehmenden Studierenden wurden schriftlich über die Studie informiert und waren mit der Teilnahme einverstanden.

### Konzept des digitalen, wissenschaftlichen Seminars

Das Seminar, bzw. die Lehreinheit (LE), begann mit einem 15-minütigen Online-Podcast (im Sommersemester) bzw. einer 15-minütigen Livepräsentation (im Wintersemester). Diese theoretische Einführung bestand darin, Kriterien guten wissenschaftlichen Arbeitens und guter wissenschaftlicher Publikationen sowie einen Leitfaden zur Erstellung eines Abstracts zu definieren. Weiterhin wurde zu den ausgewählten Publikationen des Seminars eine Einführung in die jeweiligen HNO-Krankheitsbilder gegeben.

Anschließend erfolgte eine individuelle Arbeitsphase, welche 1) darin bestand, eine um Titel, Autoren und Abstract geschwärzte wissenschaftliche Publikation zu lesen und zu verstehen und 2) hierfür einen strukturierten Abstract nach dem vorgestellten Leitfaden zu schreiben. Auf diesem Weg sollten die Studierenden eine Kernkompetenz des wissenschaftlichen Schreibens erlernen: das Verfassen eines Abstracts.

Die Publikationen wurden folgendermaßen ausgewählt: Es wurden zu zwei größeren HNO-Themenkomplexen, die auch im weiteren Semester und den Prüfungen behandelt wurden, je eine Publikation ausgewählt, um diese in der Lehre des wissenschaftlichen Seminars abzubilden. Die Publikationen hatten zum Ziel, aktuelle wissenschaftliche Fragestellungen des Faches aus diesen Themenkomplexen zu repräsentieren. Die erste Publikation wurde aus dem Bereich Otologie/Schwindel zum Stellenwert der intratympanalen Therapie bei Morbus Menière ausgewählt [23], die zweite Publikation aus dem Bereich Tumor zum Stellenwert des HPV-Status beim Kopf-Hals-Plattenepithelkarzinom [13].

### Evaluation und Studienanalyse

Die von den Studierenden verfassten Abstracts wurden auf die Online-Lehr-Plattform ILIAS [4] hochgeladen und die Evaluationen online durchgeführt. Die Originalabstracts der initial geschwärzten Publikationen wurden den Studierenden zugänglich gemacht, nachdem sie ihren eigenen Abstract hochgeladen hatten. Die Studierenden hatten die Möglichkeit, individuelles Feedback zu erhalten, indem sie den Lehrenden die Abstracts zusätzlich via E‑Mail zukommen ließen. Die Evaluation bestand aus 11 Items (E) gefolgt von 8 Basisfragen (B), die in Tab. 1 dargestellt sind. Die Items waren entweder mit Ja/Nein zu beantworten oder durch eine 6‑Punkte-Likert-Skala. Zusätzlich erfragten wir in Freitext-Kommentaren Meinungen zum Seminar und zur Bewertung einer digitalen vs. Präsenzlehre (Tab. 1).

**Table 1:** 

Item	Fragen	Antwortskala
E1a)	Betrachten Sie die LE als wertvolle Ergänzung Ihres wissenschaftlichen Arbeitens?	1: „Ich stimme absolut zu“–6: „Ich stimme überhaupt nicht zu“
E1b)	Förderte die LE Ihr Interesse an wissenschaftlichem Arbeiten? (Antwortskala von E1a)	1–6
E2)	Hätten Sie eine Präsenzveranstaltung der digitalen Lehre vorgezogen? Wieso? Oder wieso nicht?	Ja/Nein
		Freitextkommentare
E3)	Denken Sie, Sie hätten in einer Präsenzveranstaltung mehr gelernt? (Antwortskala von E1a)	1–6
E4)	Wurden Lernziele klar formuliert? (Antwortskala von E1a)	1–6
E5)	War die LE klar definiert? (Antwortskala von E1a)	1–6
E6)	Betrachten Sie den Inhalt der LE als relevant für Ihre zukünftige Tätigkeit? (Antwortskala von E1a)	1–6
E7)	Wieviel Zeit benötigten Sie für diese LE? (Seminar ist auf 60 min angesetzt)	Angabe in Minuten
E8)	Bitte bewerten Sie diese LE auf einer Skala von 1: „sehr gut“ bis 6: „insuffizient“	1–6
E9)	Bitte bewerten Sie Ihren Lernzuwachs auf einer Skala von 1: „sehr gut“ bis 6: „insuffizient“	1–6
E10)	Bitte verfassen Sie einen Freitextkommentar: Welche Verbesserungen würden Sie sich wünschen? Was mochten Sie? Was nicht?	Freitextkommentare
B1)	Alter	–
B2)	Geschlecht	–
B3)	Semester	–
B4)	Studiensemester	–
B5)	Ausbildung/Arbeitsstelle vor Studium	Ja/Nein
B6)	Wissenschaftliches Arbeiten zuvor in Studien, Postern oder auf Kongressen	Ja/Nein
B7)	Medizinische Dissertation	1: „geplant und initiiert oder begonnen“
		2: „geplant, noch nicht begonnen“
		3: „vielleicht“
		4: „nicht geplant“
B8)	Wissenschaftliche Tätigkeit/Karriere geplant	Ja/Nein

Die Evaluationen und Studienprotokolle waren auf einer Online-Plattform verfügbar und wurden nach Vervollständigung durch die Studierenden hochgeladen. Die Evaluation bestand aus elf Items (E); zusätzlich wurden acht Basis-Daten erhoben (B)

### Statistik

Die statistische Auswertung erfolgte mittels SPSS Version 27 (IBM SPSS Statistics für Windows, Version 27.0, Fa. IBM Corporation, Armonk, NY, USA). Zur Analyse der T‑Tests erfolgte vorab ein Test auf Varianzgleichheit nach Levene. Die Korrelationsanalyse erfolgte mittels Spearmans Rho. Korrelationsstärke wurde definiert als 0–0,3 (keine Korrelation), 0,3–0,5 (schwach positiv), 0,5–0,7 (moderat positiv), 0,7–0,9 (stark positiv), 0,9–1,0 (sehr stark positiv) oder entsprechend negative Korrelation. Das Level der Signifikanz wurde mit *p* < 0,05 festgelegt.

## Ergebnisse

### Studienkollektiv und wissenschaftliches Interesse

Die Tab. 2 zeigt eine Übersicht des Studienkollektivs; im Mittel befanden sich die Studierenden in Regelstudienzeit (HNO-Heilkunde wird in der Regel im achten Semester gelehrt) und waren häufiger weiblich. Mehr als ein Drittel der Studierenden arbeitete oder studierte bereits vor Eintritt in das Studium der Humanmedizin und knapp zwei Drittel hatten vor unserem wissenschaftlichen Seminar keine Erfahrung in wissenschaftlichem Arbeiten (Tab. 2). Obwohl mit ebenfalls knapp zwei Drittel die Mehrheit der Studierenden keine Ambitionen für eine wissenschaftliche Karriere angab, planten die meisten Studenten jedoch, eine medizinische Doktorarbeit zu verfassen (Tab. 1, B7 und Tab. 2), für die wissenschaftliche Fähigkeiten grundlegend notwendig sind.

**Table 2:** 

	*N* (gesamt: 265)/prozentualer Anteil
Teilnehmer	*N* = 149 Wintersemester 2020/2021
	*N* = 116 Sommersemester 2020
Alter	Mittelwert 25,38 ± 4,19 Jahre
Geschlecht	*N* = 146 (55,1 %) weiblich
	*N* = 119 (44,9 %) männlich
Studiensemester	Mittelwert 8,54 ± 2,3 Semester
Arbeit/Studium vor Aufnahme Medizinstudium	*N* = 107 (40,4 %)
Keine Vorerfahrung im wissenschaftlichen Arbeiten	*N* = 170 (64,2 %)
Keine Ambitionen für eine wissenschaftliche Karriere	*N* = 171 (64,5 %)
Planung/Vorhaben einer medizinischen Dissertation	1,78 ± 0,8 (*N* = 257)

### Evaluation: Akzeptanz und positive Bewertung trotz Bevorzugung der Präsenzlehre

**Figure 1:**
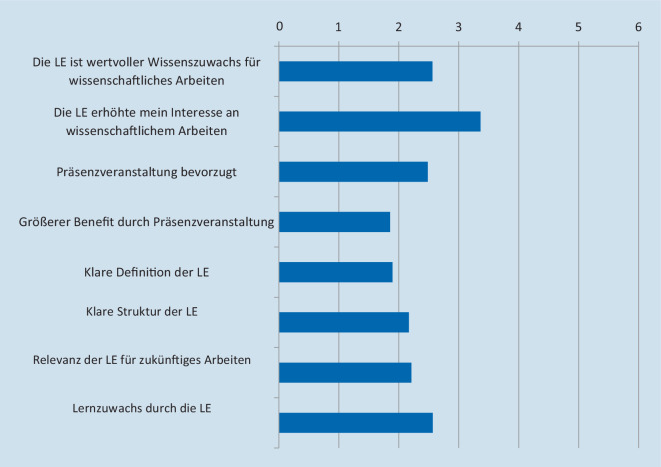

Das initiierte Seminar wurde akzeptiert und von den Studierenden positiv bewertet. Die Evaluationsitems wurden auf einer Likert-Skala erhoben (Abb. 1; 1 „Ich stimme vollständig zu“; bis 6 „Ich stimme absolut nicht zu“). Insgesamt war die LE gut bewertet mit einem durchschnittlichen Notenwert von 2,22 ± 0,88 (*n* = 261). Weiterhin wurde sie als klar strukturiert bewertet (1,89 ± 0,92, *n* = 264) und mit klarer Zielsetzung (1,86 ± 0,86, *n* = 263). Die Studierenden bewerteten die Relevanz der LE für ihre spätere berufliche Zukunft mit einer 2,17 ± 0,99 (*n* = 262) und sahen einen Lerneffekt durch die LE mit einer 2,56 ± 0,95 (*n* = 258).

Eine gute Kursevaluation korrelierte moderat mit dem beschriebenen Lerneffekt bzw. -zuwachs der LE (*p (rho):* 0,613, *p* < 0,001). Eine gering positive Korrelation konnte zwischen einer guten Evaluation der LE und zum einen der Tatsache, dass die LE gut strukturiert beschrieben wurde, festgestellt werden (*p (rho):* 0,396, *p* < 0,001), zum anderen mit der Relevanz der LE für die zukünftige berufliche Karriere (*p (rho):* 0,448, *p* < 0,001).

Die Studierenden stimmten meist zu (2,48 ± 1,06; *n* = 261), dass eine LE in Präsenz den Benefit des Lernzuwachses weiter erhöht hätte; was vor allem von männlichen Studenten so befürwortet wurde (2,3 ± 0,1 vs. 2,59 ± 0,09, *p* = 0,032). Bezüglich der zukünftigen Arbeit/Arbeitsambitionen wurde die LE als relevant bewertet (2,56 ± 1,02, *n* = 164).

### Die andauernde Pandemie führte zu einer schlechteren Bewertung des Seminars

Signifikant weniger Studierende erklärten im Wintersemester ihre Ambitionen für eine wissenschaftliche Karriere verglichen zum Sommersemester (0,77 ± 0,04 vs. 0,58 ± 0,05, *p* = 0,001). Die im Wintersemester Studierenden bewerteten die LE schlechter verglichen zu den Studierenden im Sommersemester: Lernzuwachs durch die LE (2,69 ± 0,08 vs. 2,38 ± 0,09, *p* = 0,011), Notenbewertung der LE (2,38 ± 0,08 vs. 2,01 ± 0,08, *p* = 0,001). Weiterhin bewerteten sie die LE auch als weniger relevant für ihre zukünftige Arbeit verglichen zu den Kollegen des Sommersemesters (2,29 ± 0,09 vs. 2,01 ± 0,08, *p* = 0,023). Zusammenfassend schien die andauernde Pandemiesituation die positiven Bewertungen der Studierenden für unser digitales Seminar zu vermindern, wahrscheinlich auch aufgrund wachsender Frustration durch eine anhaltend rein digitale Lehre.

### Die hohe Bearbeitungszeit spiegelte die anspruchsvolle Aufgabe der Lerneinheit wider

Die durchschnittliche Bearbeitungszeit der LE lag bei 83,88 ± 59,69 min (*n* = 238), was den erwarteten Bearbeitungsrahmen von 60 min deutlich überschritt. Dies zeigte, dass die individuelle Arbeitsphase der LE neu und herausfordernd war und der Zeitrahmen bei der Konzeptionierung primär deutlich unterschätzt worden war. Alter, Geschlecht, Studiensemester, wissenschaftliche Ambitionen, vorherige wissenschaftliche oder berufliche Tätigkeiten korrelierten nicht mit dem Bearbeitungszeitrahmen.

### Der Lernzuwachs durch die LE erhöhte das wissenschaftliche Interesse der Studierenden

Die Studierenden, welche die LE als wertvollen Zuwachs zu ihrer wissenschaftlichen Arbeit bewerteten, sahen auch ihr Interesse in wissenschaftlichem Arbeiten als gesteigert an (*p (rho):* 0,557, *p* < 0,001). Da die Mehrheit der Studierenden keine wissenschaftliche Karriere anstrebt, sah die Mehrheit auch die LE nicht als Motivationszuwachs für eine wissenschaftliche Tätigkeit an (durchschnittliche Bewertung bei 3,36 ± 1,09, *n* = 263). Nichtsdestotrotz empfanden die Studierenden mit zunehmendem Lerneffekt die LE als relevant und sahen ihr wissenschaftliches Interesse zunehmend gesteigert (*p (rho):* 0,343, *p* < 0,001).

### Freitext-Kommentare: Die Studierenden reflektierten ihre gesteigerten digitalen und wissenschaftlichen Kenntnisse

**Figure 2:**
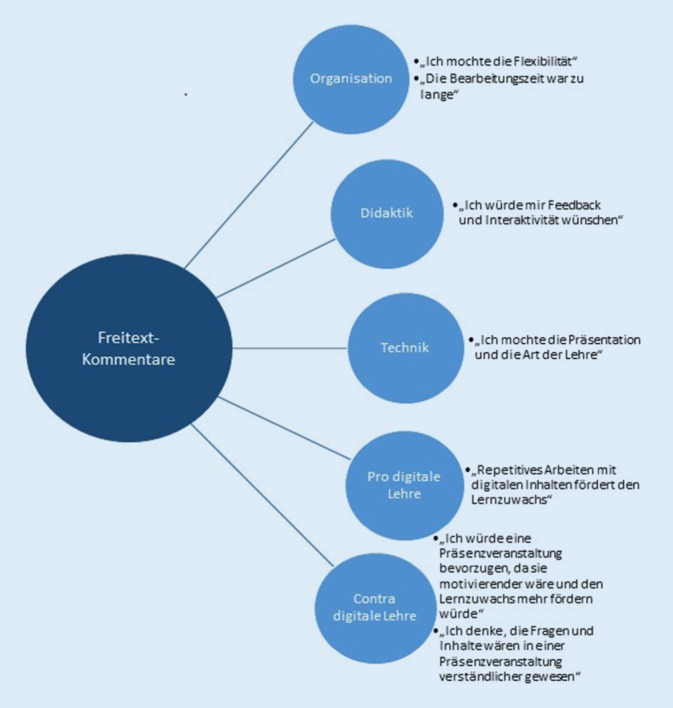

Im Folgenden wurden die wesentlichen Freitext-Kommentare der Studierenden zusammengefasst (Antwortquote 60 %; *n* = 159 Rückmeldungen/265 Teilnehmer; Zusammenfassung siehe Abb. 2): Obwohl die Studierenden den Vorteil von digitalen Lehrformaten anerkannten, sahen sie einen klaren Vorteil bei Präsenzveranstaltungen. Die digitale LE hatte den Studierenden zufolge dennoch den Vorteil von mehr Flexibilität, Selbstorganisation und Selbstverantwortlichkeit. Das Verfassen eines strukturierten Abstracts als Individualphase und das angebotene individuelle Feedback wurde sehr positiv angenommen und bewertet. Die Studierenden gaben an, dass es eine Herausforderung gewesen sei, die Originalpublikation in Gänze zu verstehen, die wesentlichen wissenschaftlichen Ergebnisse zu erkennen und diese organisiert und strukturiert wiederzugeben. Sie gaben eine Steigerung ihrer subjektiven wissenschaftlichen Kenntnisse an.

## Diskussion

Diese prospektive Fragebogen-Studie präsentiert die Evaluation von 265 Studierenden und zeigt die zufriedenstellende Implementierung eines neuen digitalen Seminars zum kompetenzbasierten wissenschaftlichen Arbeiten in der HNO-Heilkunde. Die Studierenden bestätigten, dass ihre Kompetenzen in Selbstorganisation, digitalem und wissenschaftlichem Arbeiten gefördert wurden. Die digitale Lehre selbst wurde zwar nicht ausschließlich positiv bewertet, jedoch verbanden die Studenten das Seminar als Mittel der kompetenzbasierten Lehre mit Nutzen. Die Ergebnisse der Evaluation sollten für zukünftige Weiterentwicklungen von digitalen und wissenschaftlichen Lehrformaten berücksichtigt werden.

### Digitale Lehre in der COVID-19-Pandemie: von der dringenden Notwendigkeit eines neuen digitalen Curriculums zur tragfähigen Lehre digitaler Kenntnisse

Die zunehmende Bedeutung einer Digitalisierung der Lehre wurde in der COVID-19-Pandemie besonders deutlich. Insbesondere die Tatsache, dass die medizinische Lehre v. a. in klinischen Fächern wie der HNO jedoch zwingend auf dem erfolgreichen Vermitteln praktischer Fertigkeiten beruht, macht die Herausforderungen einer Digitalisierung der Lehre deutlich. Lehrende dürfen hierbei nicht vergessen, dass Studierende in klinischen Fächern die Präsenzlehre nahezu immer bevorzugen, da der persönliche Kontakt beim Erlernen praktischer Fertigkeiten nicht vollständig digital zu ersetzen ist. Mit längerer Dauer der Pandemie werteten die Studierenden die digitale Lehre in dieser Studie zunehmend ab (Sommer- vs. Wintersemester). Am ehesten zeigte dies unserer Einschätzung nach die wachsende Frustration der Studierenden digitalen Lehrformaten im Allgemeinen gegenüber. Zusätzlich ist anzunehmen, dass die Erwartungshaltung der Studierenden digitalen Formaten gegenüber mit der Zeit seit Pandemiebeginn gestiegen war. Nichtsdestotrotz haben digitale Medizin und Lehre vor und nach der COVID-19-Pandemie auch in klinischen Fächern vermehrte Bedeutung: Deutlich wird das anhand der Entwicklung der Telemedizin, des vermehrten Einsatzes von Apps, aber auch der Entwicklungen zum Einsatz der künstlichen Intelligenz oder der robotischen Chirurgie. Die zunehmende Digitalisierung unserer Welt ist in nahezu allen Bereichen des Lebens spürbar. Die Digitalisierung der Medizin betrifft nicht nur Ärzte und medizinisches Personal, sondern auch die Patienten. Die Studierenden, welche heutzutage als die Generation der Digitalisierung verstanden werden, sind sich der Notwendigkeit digitaler Kenntnisse auch bewusst. Digitalisierung ist auch ein Ausdruck der zunehmenden Vernetzung unterschiedlicher Lehrformate, unterschiedlicher medizinischer Bereiche und von zunehmender Bedeutung der nationalen und internationalen Verknüpfung. Da die internationale Mobilität abnahm [12], wird digitale, internationale Vernetzung an Bedeutung zunehmen, und ihre Chancen sollten erkannt und genutzt werden. Inhalte digitaler Lehrformate können problemlos national und je nach Inhalt und Curriculum auch international ausgetauscht werden. Dies gilt nicht nur für die Lehre in der Medizin, sondern auch allgemein in den Naturwissenschaften. Durch die COVID-19-Pandemie wurden neue Standards der internationalen Zusammenarbeit und der Zusammenarbeit in Entwicklung und Forschung geschaffen [17]. Dennoch sind Datenkompetenz und digitale Kompetenzen noch immer nicht im nationalen Lernzielkatalog der Medizin aufgeführt. Für eine Karriere in der Medizin wird ein kompetenter Umgang mit (wissenschaftlichen) Daten als notwendig angesehen [24].

In dieser Studie reflektieren die Studierenden sowohl die Vorteile als auch die Risiken digitaler Lehre. Anhand der Freitextkommentare wird deutlich, dass sie die Digitalisierung gerade in Zeiten der aktuellen Pandemie als unvermeidbaren Prozess ansehen. Die Risiken der Digitalisierung von Lehrinhalten sind klar beschrieben: Überblick über das Thema und Strukturierung mit Zielsetzung einzelner Schwerpunkte sind schwerer zu erhalten und die Motivation ist deutlich schwerer zu vermitteln als im Präsenzunterricht [19]. Weiterhin wird die Bearbeitungszeit oft unterschätzt, und die tatsächliche Arbeitslast kann sehr schwer eingeschätzt werden [19]. Aus der Perspektive des Lehrenden ist das Verständnis für den Studierenden in digitalen Formaten schwerer aufzubringen. Aus den Freitextkommentaren in unserer Studie ging jedoch auch hervor, dass Studierende digitale Lehre mit Selbstorganisation, Selbstdisziplin und Selbstverantwortlichkeit verbinden und diese Kompetenzen zugleich als wertvoll für ihre spätere berufliche Arbeit ansehen (vgl. Abb. 2 zur Zusammenfassung der Freitextkommentare).

### Förderung von wissenschaftlichen Kenntnissen

Unsere Studie ergab, dass grundlegende wissenschaftliche Fertigkeiten durch ein fokussiertes Seminar zum digitalen wissenschaftlichen Arbeiten gefördert werden konnten. Diese Basiskompetenz bildet aus unserer Sicht die Grundlage zur Förderung digitaler Kompetenz. Das neue Konzept des wissenschaftlichen Seminars, eine wissenschaftliche Publikation zu einem ausgewählten HNO-Thema zunächst lesen und dann den geschwärzten Abstract selbst verfassen zu müssen, erforderte ein vollständiges inhaltliches und studienbezogenes Verständnis durch die Studierenden. So wurden die Studierenden zu kritischem wissenschaftlichem Lesen und fokussiertem Durcharbeiten der Publikation ermutigt, welches unserer Einschätzung nach zur Förderung ihrer wissenschaftlichen und digitalen Kompetenz beitrug. Dass die Studierenden die LE als relevant für ihre zukünftige berufliche Karriere und als Lernerfolg beschrieben, zeigte, dass die Implementierung der LE ein Erfolg war.

Unsere Auswertung ergab dabei, dass, obwohl die Mehrheit der Studierenden kein Interesse an späterer wissenschaftlicher Tätigkeit benannte, die meisten der Studierenden das Verfassen einer Doktorarbeit planten, für welche grundlegende wissenschaftliche Kenntnisse und Kompetenz notwendig sind. Der überwiegende Teil der Studierenden gab einen Lernzuwachs an. Mit zunehmendem Lernzuwachs wurde die Relevanz der LE höher bewertet. Ein gesteigertes wissenschaftliches Interesse korrelierte mit einer besseren Bewertung der LE. Das Interesse der Studierenden an wissenschaftlichem Arbeiten wurde weiter geweckt, wenn sie einen Lernzuwachs durch die LE erhielten. Dieses Ergebnis lässt umgekehrt auch die Schlussfolgerung zu, dass Studenten mit einem stärkeren Interesse an der Wissenschaft durch höhere Partizipation verstärk von der LE profitieren. Dennoch war das Feedback der LE durch die Studierenden nicht durchweg positiv, und im Durchschnitt wurde das wissenschaftliche Interesse durch die LE nicht bei allen Studierenden geweckt, was mit der geringen Anzahl der Studierenden, die eine wissenschaftliche Karriere anstreben, korreliert.

Dieses Ergebnis sehen wir als stark verbesserungswürdig an: Bei in der Zukunft der Medizin zunehmenden Überschneidungen von Wissenschaft und klinischer Tätigkeit ist die Motivation des medizinischen Nachwuchs zum wissenschaftlichen Arbeiten entscheidend. Die Möglichkeit, bereits während des Medizinstudiums wissenschaftlich tätig zu werden, muss dringend ausgebaut werden. Für eine erfolgreiche und akzeptierte Implementierung von digitalen Lehrformaten ist die Kooperation mit Studierenden von zunehmender Bedeutung [10].

Die dezentrale politische Struktur in Deutschland ist ein erschwerender Faktor der Implementierung nationaler digitaler Curricula [11]. Hier sind weitere Studien, die den Einsatz digitaler und wissenschaftlicher Lehre untersuchen, notwendig.

### Limitationen und Ausblick

Als Limitationen dieser Studie sind die Beschränkung auf eine Evaluationsstudie mit fehlender Kontrollgruppe anzusehen sowie ein fehlendes definiertes Lernziel, welches als weiteres Messinstrument hätte verwendet werden können. Die Evaluation dieser Studie ist vielmehr als erster Schritt anzusehen, um im Austausch mit den Studierenden zu unserem Ziel der kompetenzbasierten Lehre zu gelangen. Da sie die Adressaten für den Erwerb digitaler Kompetenzen und Datenkompetenz in der Medizin sind, sollte der Fokus bei der Gestaltung von Lehreinheiten auf den Bedürfnissen der Studenten liegen. Die Erfahrung der COVID-19-Pandemie kann und sollte eine ergebnisorientierte Umsetzung digitaler Lehrformate für die medizinische Ausbildung bringen. Somit können wir die folgenden Kriterien für die erfolgreiche Umsetzung digitaler wissenschaftlicher Lehre festhalten:

### Kriterien für die erfolgreiche Implementierung von digitaler wissenschaftlicher Lehre

- Durchdachte Struktur sowie gesicherte personelle und finanzielle Grundlage für die digitale Umsetzung
- Kontinuierliche Evaluation und Kooperation mit den Studierenden
- Ausgewogene Implementierung von digitalen Formaten und Präsenzveranstaltungen
- Ergebnis- und kompetenzorientierter Einsatz der Lehre mit dem Fokus auf relevanten digitalen und wissenschaftlichen Kompetenzen für den medizinischen Nachwuchs

## Fazit für die Praxis

- Digitale wissenschaftliche Lehre kann in einem Seminar mit dem Fokus auf ausgewählte HNO-Krankheitsbilder und Publikationen eingesetzt werden.
- Die Studierenden bewerteten trotz ihres vorrangigen Wunsches nach Präsenzlehre das neue digitale wissenschaftliche Seminar als positiv und gewinnbringend.
- Das Seminar erfüllte die Ansprüche von kompetenzbasierter Lehre in der Medizin sowohl durch die Vermittlung digitaler als auch wissenschaftlicher Kenntnisse.
- Das rückwärts gerichtete Design der Lehreinheit (LE) förderte die wissenschaftliche Kompetenz der Studierenden, indem zuerst Kriterien guter wissenschaftlicher Publikationen gelehrt wurden und anschließend ausgewählte Publikationen verstanden und ein strukturierter Abstract verfasst werden musste.
- Die erfolgreiche Lehre digitalen wissenschaftlichen Arbeitens ist notwendig, um wissenschaftliches Interesse zu steigern und zugleich mehr wissenschaftlichen Nachwuchs zu rekrutieren.
